# Characteristics of leukemic stem cells in acute leukemia and potential targeted therapies for their specific eradication 

**DOI:** 10.20517/cdr.2021.140

**Published:** 2022-05-05

**Authors:** Quinty Hansen, Costa Bachas, Linda Smit, Jacqueline Cloos

**Affiliations:** Department of Hematology, Amsterdam UMC, Location VUmc, Cancer Center Amsterdam, Amsterdam 1081HV, The Netherlands.

**Keywords:** Acute myeloid leukemia, acute lymphoid leukemia, leukemic stem cells, targeted therapy

## Abstract

In acute myeloid leukemia (AML), a small cell population that contains stem cell features such as lack of differentiation, self-renewal potential, and drug resistance, can be identified. These so-called leukemic stem cells (LSCs) are thought to be responsible for relapse initiation after initial treatment leading to successful eradication of the bulk AML cell population. Since many studies have aimed to characterize and eliminate LSCs to prevent relapse and increase survival rates of patients, LSCs are one of the best characterized cancer stem cells. The specific elimination of LSCs, while sparing the healthy normal hematopoietic stem cells (HSCs), is one of the major challenges in the treatment of leukemia. This review focuses on several surface markers and intracellular transcription factors that can distinguish AML LSCs from HSCs and, therefore, specifically eliminate these stem cell-like leukemic cells. Moreover, previous and ongoing clinical trials of acute leukemia patients treated with therapies targeting these markers are discussed. In contrast to knowledge on LSCs in AML, insight into LSCs in acute lymphoid leukemia (ALL) is limited. This review therefore also addresses the latest insight into LSCs in ALL.

## INTRODUCTION

Acute leukemia is a rapidly progressing hematological malignancy that causes more than ten thousand deaths per year in the United States alone^[1]^. Acute leukemia can be subdivided into two main classes: acute myeloid leukemia (AML) and acute lymphoid leukemia (ALL). AML is a heterogeneous malignancy characterized by the proliferation and accumulation of myeloid progenitor cells in the bone marrow (BM) and peripheral blood^[1,2]^, while in ALL lymphoid progenitor cells accumulate in the BM and peripheral blood^[3]^. Based on the immunophenotype, ALL can be subdivided into different types; B-cell acute lymphoid leukemia (B-ALL) and T-cell acute lymphoid leukemia (T-ALL), with B-ALL being the most common type^[4]^. The median age of onset differs between AML and ALL. While ALL is most commonly diagnosed in children, AML occurs most frequently in patients older than 65 years of age^[1,5]^. Despite standard treatment with intensive cytotoxic induction chemotherapy and various clinical trials, five-year overall survival (OS) rates remain poor, especially in adults^[6-8]^. For treated AML patients between 60 and 70 years old, the OS rate is about 25%, and for ALL patients aged 70 years and older, it is only 15%. This is much lower compared to OS rates in children: around 80% in ALL and 70% in AML, depending on the risk group classification^[9,10]^. A major cause of dismal outcomes for both AML and ALL patients is the high relapse rate^[7,8]^. The prevention of relapse remains one of the most complicated challenges in the treatment of acute leukemia. However, the discovery of a rare so-called leukemia stem cell (LSC) population in AML has led to more insight into the mechanisms of relapse development and thereby novel therapeutic opportunities.

In the 1990s, a detailed investigation of AML subpopulations provided the first proof of a rare LSC population, with a CD34+/CD38- phenotype, within AML capable of establishing leukemia in nonobese diabetic/severe combined immunodeficiency (NOD/SCID) mice: leukemic stem cells (LSCs)^[11,12]^. This subpopulation of cells shares several properties with normal hematopoietic cells (HSCs). For example, AML LSCs are, similar to HSCs, self-renewing cells that remain undifferentiated themselves but are capable of giving rise to both a stem cell copy and more differentiated progeny cells through mitotic cell division^[13]^. In addition, direct evidence shows that AML consists of three distinct LSC classes with heterogeneity in their self-renewal potential: short-term, long-term, and quiescent long-term LSCs^[14]^. Since HSC compartments have a similar hierarchical structure of heterogeneous cell classes, it is indicated that LSCs in AML originate from normal HSCs^[12]^. In concordance with these functional characteristics, it is suggested that AML LSCs share specific stem cell transcriptional programs with HSCs^[15]^. Altogether, this provides strong evidence for a hierarchical organization in AML with LSCs at the apex. Only these LSCs have the ability to initiate and fuel the disease, distinguishing them from more differentiated non-tumorigenic leukemic cells and healthy cells^[13,16]^. The stem cell features that distinguish LSC from healthy cells or more differentiated leukemic cells not only provide the capacity to initiate and maintain leukemia but are also thought to contribute to relapse. For example, drug-resistant properties due to changes in the expression of drug resistance genes are attributed to the stem cell phenotype^[13,17-19]^. The clinical relevance of LSCs is underlined by studies that showed an increased chance of relapse and worse overall survival in AML patients with a high CD34+/CD38- LSC frequency at diagnosis and after induction therapy compared to patients with a low LSC frequency^[20]^. These data suggest that eliminating LSCs during or after induction therapy will be crucial in improving the clinical outcome of AML patients.

Besides functional similarities, LSCs and HSCs also differ in many characteristics, such as cell surface protein expression or activation of intracellular signaling pathways, which may be exploited to specifically eliminate LSCs while sparing HSCs. Some of the key signaling pathways that play a role in the regulation of self-renewal, survival, proliferation, and differentiation are dysregulated in LSCs *vs.* HSCs. Examples of such signaling pathways include JAK/STAT, Wnt/β-catenin, Hedgehog, and Notch^[21-24]^. The dysregulation of these key signaling pathways in LSCs not only contributes to their oncogenic potential and cancer progression, but some of them also contribute to drug resistance, illustrating the importance of therapeutically targeting these pathways.

Other factors suggested to be involved specifically in drug resistance of LSC and therefore promising targets are intracellular enzymes such as aldehyde dehydrogenase (ALDH) and histone deacetylase (HDAC)^[25,26]^. In addition, the overexpression of drug efflux transporters such as ATP-binding cassette transporters is also suggested to be an important intrinsic resistance mechanism (comprehensively reviewed in^[27]^), but treatment with specific inhibitors remains controversial for acute leukemia. Recently, there has been a revival of the research into the metabolic rewiring of resistant cancer cells. For LSCs specifically, the pathway of mitochondrial oxidative phosphorylation seems to be very distinct, as comprehensively reviewed by de Beauchamp *et al.*^[28]^. Extrinsic factors, including proteins involved in the cell-to-cell interactions between LSCs and the tumor microenvironment, could sensitize LSCs for eradication^[29,30]^. In addition to these intrinsic and extrinsic mechanisms, identification of aberrantly expressed surface markers on LSCs in AML is also a technique of interest in order to specifically eradicate these cells. Due to surface markers uniquely expressed on AML LSCs and not on HSCs, specific drug delivery to the rare population of LSCs is feasible without harming normal stem cells^[31,32]^.

While knowledge on LSCs in AML has rapidly increased over the past few years, less is known about the nature of LSCs in ALL. There are even contradictory results on the existence of an LSC population in ALL. Several studies support a similar hierarchical organization in ALL as AML, while other studies provide evidence suggesting that the stochastic model may fit better^[33,34]^. This model states that all tumor cells are biologically equal and have the same tumor initiating potential. Their intrinsic characteristics cannot predict their behavior, so enrichment of these cells by sorting them based on these characteristics is impossible according to this model^[16]^. Therefore, besides summarizing the most important AML LSC specific markers and recent clinical trials targeting these markers, evidence and important markers of a stem cell population in ALL are also discussed.

## LEUKEMIC STEM CELL TARGETS IN ACUTE MYELOID LEUKEMIA

### AML LSCs cell surface protein targets

Similar to what is seen for AML LSCs, HSCs also have a CD34+/CD38- phenotype. The challenge in eliminating LSCs while sparing HSCs is therefore to find unique markers on LSCs that distinguish them from HSCs. Several studies have shown markers that are aberrantly expressed on LSC but not or very lowly expressed on normal HSC^[32,35]^. These markers can be used to isolate LSCs for further characterization, as well as monitored during therapy^[20]^. In addition, these markers are investigated for specific targeting by therapeutics, such as antibody-drug conjugates (ADCs) and chimeric antigen receptor (CAR)-T cells directed to these LSC-specific markers. The most important LSC specific cell surface markers are detailed below.

#### CD123

One of the surface markers that has been shown to be overexpressed in primary AML blasts and LSCs is CD123 [interleukin-3 receptor alpha (IL-3Rα)]. CD123 expression was not detectable on normal CD34+/CD38- hematopoietic cells, HSCs, discriminating LSCs from HSCs^[36]^. Despite the lack of CD123 expression on HSCs in healthy subjects, several other studies did find CD123 expression on HSCs and more differentiated hematopoietic cells such as monocytes^[37,38]^. For instance, NOD/SCID mice injected with patient cells from cord blood and BM revealed that not only human AML LSCs expressed CD123, but also a small proportion of normal human HSCs were positive for CD123^[37]^. However, since the percentage of CD123+ HSCs in the bone marrow was relatively low and only a relatively small part of HSCs expressed CD123, most HSCs should not be harmed by CD123-targeted therapy^[37]^.

Elevated levels of CD123 in AML are correlated to an increased number of leukemic blasts at diagnosis, a decreased chance to achieve complete remission and poor survival rates^[39]^. Moreover, high CD123 expression was associated with more cell cycle activity in the leukemic blasts, apoptotic resistance, and elevated signal transducer and activator of transcription 5 (STAT5) activation by IL-3^[39]^. The biological basis explaining this poor survival, when blasts have high CD123 expression, is enhanced signaling via the IL-3R in the CD123 overexpressing AML LSCs, resulting in increased proliferation and cell viability and decreased CXCR4 expression^[30]^. CXCR4 is a receptor expressed by HSCs/LSCs that interacts with stromal-derived factor 1 (SDF1), a chemokine constitutively expressed by BM stromal cells^[40]^. This interaction plays a major role in the homing and preserving of the stem cells in the BM niche. Downregulation of CXCR4 *in vitro* impaired the migration of LSCs to SDF1, suggesting that high CD123 expression and downregulated CXCR4 in LSCs releases them from the chemoprotective BM niche into the circulation^[30]^. A promising strategy to eliminate CD123-expressing AML LSCs could therefore be to combine CXCR4 antagonists with CD123 antibodies, since the antagonists would release more cells from the BM, leading to more effective targeting of LSCs by CD123 antibodies.

#### CD33

Another surface marker identified on LSCs is CD33, also known as sialic acid-binding Ig-like lectin 3^[37]^. Since CD33 shows similar homogeneous expression in relapsed AML samples as CD123, it was suggested that both these surface markers are promising drug targets^[38]^. Gemtuzumab ozogamicin (GO), a humanized anti-CD33 monoclonal antibody attached to a cytotoxic agent, has been used in the clinic and has shown clinical efficacy in the treatment of AML. However, since CD33, similar to CD123, shows expression on HSCs, healthy BM myeloid progenitor cells, and more differentiated myeloid cells, the risk of unwanted on-target off-tumor toxicity increases after targeting these molecules^[38]^. This is especially seen in ADCs targeting CD33, since the expression of CD123 is more restricted in healthy BM cells compared to CD33 expression^[41]^. In addition, the clinically effective GO revealed severe toxicities such as liver and hematological toxicities^[41,42]^. Therefore, targeting of more specific surface markers is required to reduce such toxicities.

#### T cell immunoglobulin mucin-3

T cell immunoglobulin mucin-3 (TIM-3), a transmembrane protein initially found on differentiated CD4+ Th1 and CD8+ Tc1 cells, is a membrane marker expressed on AML cells^[43]^. TIM-3 is expressed on multiple immune cells, such as regulatory T cells, natural killer cells, dendritic cells, monocytes, and macrophages^[44-46]^. Transcriptional profiling of LSCs and HSCs derived from human AML samples showed that TIM-3 is highly expressed on most LSCs, with the exception of acute promyelocytic leukemia LSCs, but not expressed on normal HSCs^[47]^. In addition, only TIM-3-positive AML cells, and not TIM-3 negative ones, were able to regenerate AML in immune-deficient mice. A similar differential expression of TIM-3 between LSCs and HSCs was observed in a study performing flow cytometry on primary human AML samples^[48]^. This differential expression allows for prospective separation of LSCs from normal HSCs, and it is also promising for the successful elimination of LSCs in AML^[48]^. Moreover, the number of TIM-3 expressing LSCs after allogeneic stem cell transplantation seems to be predictive for relapse^[49]^.

That LSCs can effectively be eliminated by targeting TIM-3 is supported by *in vivo* experiments in human AML xenograft mice using the anti-human TIM-3 mouse antibody named ATIK2a. This antibody induces antibody-dependent cell-mediated cytotoxicity and complement-dependent cytotoxicity, which resulted in the effective eradication of LSCs without harming HSCs. The outcome of this was a strong decrease in leukemic burden in treated mice^[47]^. Altogether, these studies provide evidence that TIM-3 is a promising target in the elimination of LSCs and suggest that targeting of TIM-3 results in fewer side effects compared to the targeting of, e.g., CD33.

#### C-type lectin-like molecule-1

C-type lectin-like molecule-1 (CLL-1), a transmembrane glycoprotein, was first identified on AML cells in 2004^[50]^. CLL-1 is expressed in 92% of primary AML samples^[50]^ and expressed on LSCs in the majority of AML patients, but it is absent on HSCs from healthy and regenerating BM from patients who received chemotherapeutic treatment^[51,52]^. It was recently published that CLL-1 expression can also be bimodal in AML samples^[53]^, which warrants further investigation into effective elimination of LSCs including those negative for CLL-1. Despite this, CLL-1 is still a promising target in the treatment of AML. Zheng *et al.*^[54]^ studied the efficacy and safety of an anti-CLL-1-ADC in which a CLL-1 antibody is conjugated via a self-immolative disulfide linker to a pyrrolobenzodiazepine (PBD) dimer. Xenograft mice and cynomolgus monkeys were treated with this anti-CLL-1 ADC and showed an effective decrease in AML cells. Despite the fact that CLL-1 is expressed on healthy myeloid progenitors, its expression pattern is more restricted on healthy cells than that of CD33, resulting in less toxicity and faster recovery from side effects such as cytopenia^[54]^. Anti-leukemic effects were also observed in xenografted mice engrafted with human AML and treated with CAR-T cells targeting CLL-1^[52]^. The results show both* in vitro* and *in vivo* compelling anti-leukemic effects.

#### CD47

CD47 is a surface marker widely expressed on both hematopoietic cells and other cell types^[55]^. The interaction between CD47 and SIRPα, a protein expressed on phagocytic cells such as dendritic cells and macrophages, leads to inhibition of phagocytosis^[56]^. The CD47 surface marker is, compared to the other mentioned surface markers, not as stem cell-specific and therefore not used to identify and monitor stem cells. However, since it was found that CD47 has elevated expression on AML LSCs compared to normal HSCs, blocking monoclonal antibodies have been used in multiple studies as a strategy to eliminate LSCs^[56,57]^. The results show that these blocking anti-CD47 antibodies enable phagocytosis, resulting in the eradication of LSCs without affecting normal cells^[55-57]^.

Targeting LSCs via one protein is a big challenge, since even a low expression of a cell surface marker on HSCs leads to unwanted toxicities; thus, more specific targeting of LSCs is required to prevent this. One option to increase efficacy and decrease toxicity is by targeting surface marker combinations that are highly co-expressed on AML cells but not co-expressed on healthy cells. Haubner *et al.*^[38]^ showed that this is valid for the CD33/TIM-3 and CLL-1/TIM-3 combinations.

Different therapies have been developed to target these surface markers specifically expressed on LSCs [Table 1]. Besides the described surface markers, other commonly investigated surface markers are also included in the table. 

**Table 1:** Examples of recently developed therapies against AML LSC surface markers

**Therapy type**	**Surface marker**	**Drug name**	**Ref.**
*Monoclonal antibodies*			
Naked antibody 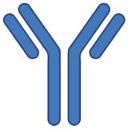	CD123 CD33 TIM-3 CD96 CD99 CD44 CD47	CSL362 KHK2823 Lintuzumab ATIK2a MBG453 MSH-TH111e H036-1.1 H90 B6H12.2 BRIC126 Hu5F9-G4 CC-90002	Busfield *et al.*^[58]^ 2014 Akiyama *et al.*^[59]^ 2015 Sutherland *et al.*^[60]^ 2009 Kikushige *et al.*^[47]^ 2010 Schürch^[61]^ 2018 Gramatzki *et al.*^[62]^ 2016 Chung *et al.*^[63]^ 2017 Gadhoum *et al.*^[64]^ 2016 Majeti *et al.*^[56]^ 2009 Liu *et al.*^[57]^ 2015 Narla *et al.*^[55]^ 2017
Antibody-drug conjugate 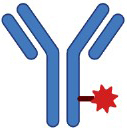	CD123 CD33 CLL-1 CD25	IMGN632 SGN-CD123A Gemtuzumab ozogamicin (MyloTarg®) SGN-CD33A Anti-CLL-1-ds-PBD ADCT-301	Kovtun *et al.*^[65]^ 2018 Li *et al.*^[66]^ 2018 Gottardi *et al.*^[67]^ 2020 Kung Sutherland *et al.*^[68]^ 2013 Zheng *et al.*^[54] ^2019 Flynn *et al.*^[69,70]^ 2014, 2016
Radioimmunoconjugate 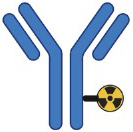	CD123 CD33	111In-DTPA-NLS-CSL360 225Ac-lintuzumab	Gao *et al.*^[71]^ 2016 Jurcic *et al.*^[72]^ 2013
*Bispecific antibodies*			
Dual-affinity re-targeting antibody (DART) 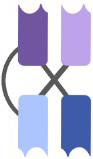	CD123xCD3	Flotetuzumab	Chichili *et al.*^[73]^ 2015
Bi-specific T-cell Engager (BiTE) 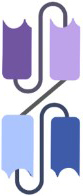	CD123xCD3 CD33xCD3	BiTE(CSL263/OKT3) AMG-330 AMG-673	Hutmacher *et al.*^[74]^ 2019 Krupka *et al.*^[75]^ 2014 Subklewe *et al.*^[76]^ 2019
T cell-dependent bispecific (TDB) antibody 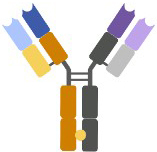	CLL-1xCD3	CD3/CLL-1 TDB MCLA-117	Leong *et al.*^[77]^ 2017 van Loo *et al.*^[78]^ 2019
Other bispecific antibodies	CD47xCD33	HMBD004	Boyd-Kirkup *et al.*^[79]^ 2017
*Chimeric antigen receptor (CAR)-T cells*			
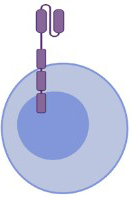	CD123 CD33 CLL-1 CD44v6 CD7	MB-102 CART-33 CLL-1 CAR-T cells CD44v6.CAR28z CD7 CAR-T cells	Mardiros *et al.*^[80]^ 2013 Kenderian *et al.*^[81]^ 2015 Wang *et al.*^[52]^ 2018 Casucci *et al.*^[82]^ 2013 Gomes-Silva *et al.*^[83]^ 2019
*Other*			
TRAIL	CLL-1 CD25	CLL-1:TRAIL IL2-TRAIL	Wiersma *et al.*^[84]^ 2015 Madhumathi *et al.*^[85]^ 2017
SAR-transduced T cells 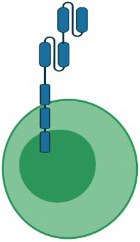	CD123 CD33	Anti-E3-anti-CD123 taFv Anti-E3-anti-CD33 taFv	Benmebarek *et al.*^[86]^ 2021 Benmebarek *et al.*^[86]^ 2021

Figures created with BioRender.com. SAR: Synthetic agonistic receptor; TRAIL: tumor necrosis factor-related apoptosis-inducing ligand; CD44v6: CD44 variant domain 6; taFv: tandem single chain variable fragment.

The surface markers in Table 1 are all surface markers of AML LSCs that have been investigated by many different studies. Recently, two novel and less thoroughly investigated candidate AML LSC surface markers have been identified. First, CD9, a member of the tetraspanin family, was found to be highly expressed on LSCs in AML patients, and CD9-positive AML cells were capable of initiating AML *in vivo*^[87,88]^. Since CD9 is hardly expressed on normal HSCs, it was suggested that this surface marker could be a promising new target for the eradication of LSCs. Second, AML cells positive for c-MPL, a thrombopoietin receptor regulating processes such as self-renewal and HSC proliferation, showed more chemoresistance than the c-MPL negative AML cell population in a mouse leukemic model^[89]^. Moreover, the c-MPL+ cells had a higher self-renewal potential and were significantly better at initiating AML *in vivo* compared to the c-MPL- cell population. Although these results suggest that c-MPL could be a potential target to eradicate AML LSCs, more research is needed regarding unwanted toxicities since c-MPL is also a long-term HSCs marker^[90]^.

### Signal transduction pathways and transcription factors involved in AML LSC survival

Besides the previously described surface markers, there are intracellular proteins such as transcription factors that are differentially expressed between LSCs and HSCs^[91]^. Several of these factors are involved in drug resistance mechanisms, making them important therapeutic targets for elimination of AML LSCs.

#### JAK/STAT signaling pathway

**Figure 1:**
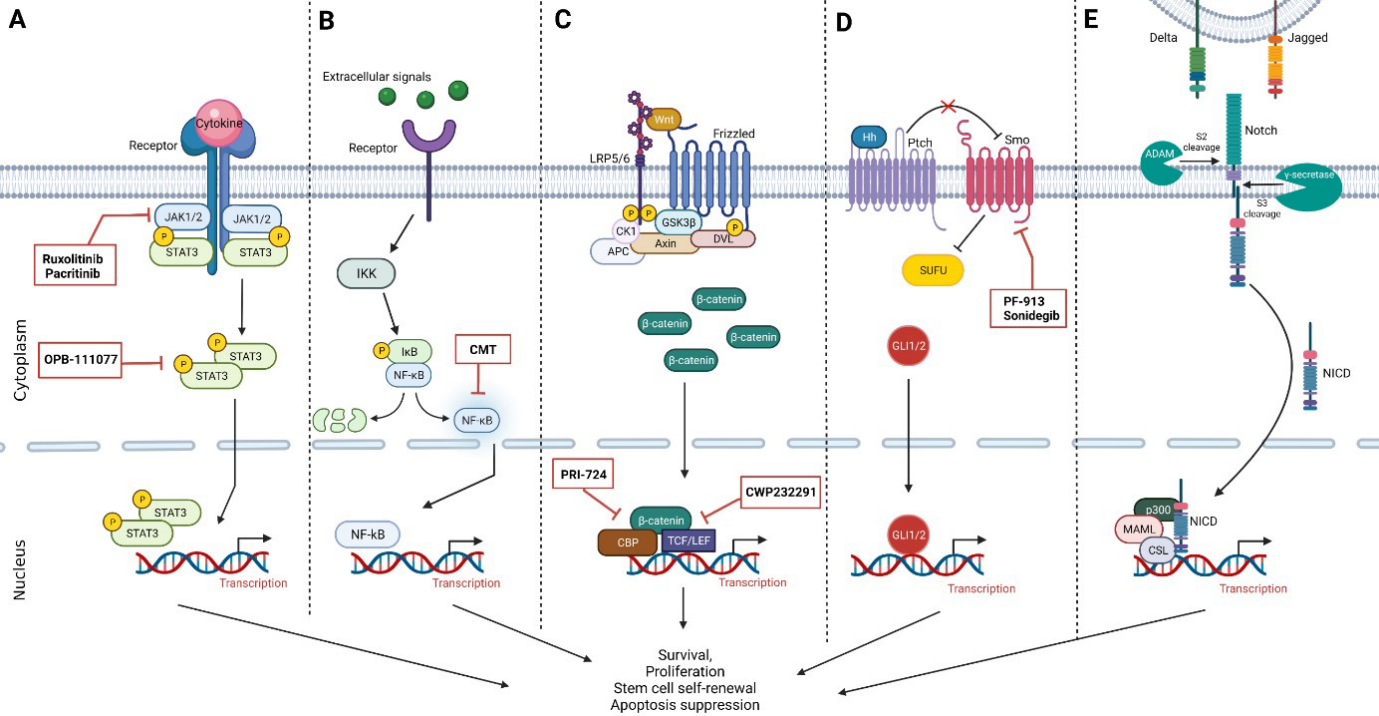
Intracellular signaling pathways dysregulated in AML LSCs, including agents recently used in clinical trials inhibiting pathway activity. (A) JAK/STAT signaling pathway. Therapeutic agents either target elevated JAK1/2 levels or constitutively active STAT3. (B) NF-κB signaling pathway. Therapeutic agents recently used in clinical trials directly inhibit the constitutively activated NF-κB. (C) Wnt/β-catenin signaling pathway. Therapeutic targets prevent constitutive activation by inhibiting the interaction between β-catenin and CBP or TCF. (D) Hh signaling pathway. Small molecule inhibitors target Smoothened (Smo), decreasing pathway activation. (E) Notch signaling pathway. There are currently no clinical trials inhibiting or activating the Notch pathway as a treatment in AML patients. Created with BioRender.com. AML: Acute myeloid leukemia; LSCs: leukemic stem cells; JAK/STAT: Janus kinase/signal transducer and activator of transcription; NF-κB: nuclear factor-kappa B; CBP: CREB-binding protein; TCF: T-cell factor; Hh: Hedgehog.

High CD123 expression is associated with elevated STAT5 activation by IL-3, suggesting that the Janus kinase/signal transducer and activator of transcription (JAK/STAT) signaling pathway plays a role in the fate of LSCs [Figure 1A]^[39]^. The JAK family of intracellular non-receptor tyrosine kinases can be activated via extracellular cytokine or growth factor binding, resulting in the phosphorylation and activation of STAT proteins^[92]^. This STAT protein family consists of transcription factors that interfere with proliferation, differentiation, and apoptosis. Previous research has shown that STAT3 and STAT5 are constitutively activated in AML leukemic blasts, which is not seen in HSCs^[93]^. This may contribute to the uncontrolled proliferation of these blasts and resistance to chemotherapy-induced apoptosis. The role of JAK/STAT signaling in AML LSCs growth and survival was investigated several years later by evaluating the expression levels of JAK and STAT in AML patient samples at diagnosis and relapse^[94]^. An ATP competitive inhibitor of JAK1/2 kinases named AZD1480 was used in both *in vitro* and* in vivo* experiments to analyze its effect on AML stem/progenitor cells. Inhibitor treatment of AML CD34+ cells *in vitro *showed decreased levels of JAK2 and STAT3/5 activity and reduced AML CD34+ cell proliferation and survival, but it did not affect normal CD34+ cells^[94]^. Similar results were seen in NOD/SCID mice treated with AZD1480: the number of AML LSCs was reduced, but normal human HSC numbers were not affected. To further investigate the role of JAK1, JAK2, STAT3, and STAT5 in CD34+ AML cells, an RNA interference-mediated knockdown of these proteins was performed. JAK2, STAT3, and STAT5 knockdown resulted in a significant decrease of colony-forming cells, cell numbers, and survival, while this was not observed with downregulation of JAK1, indicating that inhibition of JAK2 is more effective in decreasing growth and survival of AML CD34+ cells than inhibition of JAK1.

#### Nuclear factor-kappa B signaling pathway

Nuclear factor-kappa B (NF-κB) is a proinflammatory transcription factor that plays an essential role in cellular processes such as proliferation, survival, stress responses, and inflammation^[95]^. NF-κB is suggested to be involved in drug resistance, as NF-κB has anti-apoptotic activity and increased levels of NF-κB have been seen after chemotherapy and radiotherapy^[96,97]^. Electrophoretic mobility shift assays on primary AML samples showed constitutively activated NF-κB in AML LSCs. In contrast, in normal human stem/progenitor cells, there was no NF-κB expression^[23]^. Multiple studies have investigated the effect of NF-κB inhibitors on AML (stem) cells. For example, AML LSCs treated with MG-123, an NF-κB inhibitor, initiated cell death *in vitro*^[23]^. Micheliolide (MCL), a natural sesquiterpene lactone, had cytotoxic effects on LSCs via the inhibition of NF-κB^[98]^. *In vitro *MCL treatment initiated apoptosis in AML LSCs but not in normal HSCs. *In vivo*, treatment with DMAMCL, the pro-drug form of MCL, improved survival rates of NOD/SCID mice engrafted with human AML^[98]^. A third NF-κB inhibitor, BMS-345541, conferred an altered expression of genes involved in a previously described 17-gene LSC score to primary AML patient samples^[15,95]^. This gene signature contains 17 stemness-related genes differentially expressed between LSC-positive and LSC-negative AML samples. The NF-κB pathway is illustrated in Figure 1B, including drugs recently used in clinical trials targeting this pathway. In addition, it has to be noted that this pathway may also be linked to the PI3K/Akt/mTOR pathway, which has also been suggested to be involved in the drug resistance of AML LSC^[99]^.

#### Wnt/β-catenin signaling pathway

The Wnt/β-catenin signaling pathway is a highly conserved signaling pathway involved in the development and tissue homeostasis^[100]^. The Wnt/β-catenin pathway plays a role in the cellular processes of HSCs, including cell proliferation, differentiation, survival, and stem cell renewal^[101]^. The transcriptional coactivator β-catenin plays a central role in this pathway; when the Wnt ligand binds to its cognate receptor Frizzled, β-catenin degradation is blocked, causing an accumulation of β-catenin and subsequent translation to the nucleus. There, β-catenin binds to nuclear transcription factors that belong to the T-cell factor/lymphoid enhancer factor (TCF/LEF) family, which recruits coactivators including the CREB-binding protein (CBP), resulting in the transcription of target genes involved in self-renewal and proliferation^[102]^. A study evaluating the activity of Wnt/β-catenin signaling by transfecting AML and normal progenitors with a TCF/LEF reporter construct, found that in the majority of the AML samples, the TCF/LEF pathway was constitutively active, which was supported by other studies showing overexpression of β-catenin both in AML cell lines and patient samples^[101,103]^. Moreover, the promoter regions of Wnt pathway inhibitor genes were frequently methylated in cell lines, and in 89% of AML patient samples with normal cytogenetics one or more of these inhibitor genes were methylated. Methylation of two Wnt pathway inhibitor genes named *sFRP2* and *sFRP5* was associated with elevated relapse risk, suggesting that enhanced Wnt activity has adverse outcomes in AML patients with normal karyotypes^[104]^.

Wang *et al.*^[105]^, using an AML mouse model, showed that LSCs require β-catenin in order to maintain their self-renewal capacity. Several studies have shown the overexpression of β-catenin in LSCs; however, the expression of β-catenin in normal HSCs was also observed^[106]^. Cobas *et al.*^[107]^ provided evidence that β-catenin did not show to be crucial for self-renewal of adult HSCs, since depletion of β-catenin in mice showed no impairment in hematopoiesis and lymphopoiesis. Targeting β-catenin is therefore suggested to be promising in the eradication of LSCs, while sparing HSCs [Figure 1C]. Nevertheless, targeting the Wnt/β-catenin pathway remains a challenge due to its complexity. First, mammals contain 19 different Wnt ligands and over 15 Frizzled receptors and co-receptors. In addition, targeting β-catenin is also complex since it can bind to many other transcription factors besides TCF/LEF^[108]^. Numerous small molecule inhibitors or antagonists with different targets within the Wnt/β-catenin pathway have been investigated, including inhibitors targeting the interaction between β-catenin and TCF^[109]^. Treatment with these inhibitors and antagonists is indicated to be relevant in LSC depletion by impairing their self-renewal capacity.

#### Hedgehog and Notch signaling pathways

Besides the Wnt/β-catenin pathway, the Hedgehog (Hh) and Notch signaling pathways are also highly evolutionarily conserved pathways involved in the development and tissue homeostasis^[110]^. A study in zebrafish treated with a Hh inhibitor suggested that Hh signaling is necessary for HSC homeostasis and differentiation^[111]^. A few years later, a study addressed Hh function specifically in adult HSCs, revealing that the deletion or overexpression of Smoothened, a G-protein coupled receptor playing a key role in Hh signaling, did not affect adult HSC self-renewal *in vivo*^[112]^. Studies investigating Notch signaling in HSCs showed controversial results as well. Notch signaling is activated in HSCs, but it decreases when HSCs differentiate. Inhibition of Notch signaling demonstrated increased HSC differentiation *in vitro* and depleted HSC levels *in vivo*, indicating that Notch signaling is essential for the self-renewal of HSCs^[113]^. This is contradictory to the results of follow-up studies that showed that inhibition of Notch signaling had no effect on HSCs^[114,115]^.

Aberrant Notch and Hh signaling has been detected in AML LSCs. LSCs that exhibited active Hh signaling showed enhanced survival and chemoresistance^[116]^. This, together with the knowledge that inhibiting Hh does not affect HSCs^[112]^, suggests that targeting Hh in AML patients could specifically eliminate AML LSCs [Figure 1D]. This idea was supported by a study that showed induction of apoptosis in CD34+ leukemic cells after treatment with a Hh neutralizing antibody or Smoothened antagonist^[116]^. Besides Hh signaling, the role of Notch signaling has also been investigated in AML, showing controversial results. Depending on the context, Notch signaling could exhibit both oncogenic and tumor suppressor functions *in vivo*^[117]^. In AML, Notch signaling has been shown to be mainly tumor suppressive. For instance, CD34+/CD38- LSCs, harvested from an MLL-AF9-driven AML mouse model, contained silenced Notch activity^[118]^. Both* in vivo* and *in vitro*, activation of the Notch pathway, by a gain of function models or treatment with a Notch ligand, respectively, led to decreased proliferation and increased apoptosis of this CD34+/CD38- LSC population^[118]^. However, despite several studies showing a tumor suppressive role of Notch signaling in AML LSCs, there were also studies providing evidence for an oncogenic role. For example, an oncogenic role of Notch signaling was observed in a pre-leukemic acute promyelocytic leukemia mice model^[119]^. In addition, crosstalk between the Wnt/β-catenin and Notch pathways displayed a promoting role of Notch signaling in AML development^[120]^. When β-catenin is activated, osteoblasts in the BM start to express the Notch ligand Jagged-1, resulting in activated Notch signaling in pre-leukemic hematopoietic stem/progenitor cells^[120]^. This led to the malignant transformation of these cells, providing evidence that activated Notch has an oncogenic function. However, in contrast to these results, constitutive low Notch and high Wnt signaling in LSCs was demonstrated to play a role in maintaining AML^[121]^. This result suggests that promoting Notch signaling while blocking Wnt signaling could be a promising approach to eliminate LSCs in AML. The Notch signaling pathway and a promising therapeutic agent recently used in clinical trials is shown in Figure 1E.

In addition to the described signaling pathways, there are many other factors identified as being promising LSC targets, including miRNAs^[122]^. Examples of the most important markers and therapeutics targeting them are shown in Table 2.

**Table 2:** Examples of recently developed drugs targeting differentially expressed intracellular pathways and other factors in AML LSCs

**Marker**	**Target**	**Drug type**	**Drug name**	**Ref.**
*Signaling pathways*				
JAK/STAT	JAK1/2 JAK1/2 JAK2 STAT3 STAT3 STAT3 STAT5	ATP competitive inhibitor ATP competitive inhibitor Small molecule inhibitor Antisense oligonucleotide Small molecule inhibitor dODN competitive inhibitor SH2 domain inhibitor	AZD1480 Ruxolitinib Pacritinib AZD9150 OPB-111077 CpG-STAT3dODN AC-4-130	Cook *et al.*^[94]^ 2014 Cook *et al.*^[94]^ 2014 Balaian *et al.*^[123]^ 2016 Shastri *et al.*^[124]^ 2018 Wilde *et al.*^[125]^ 2019 Zhang *et al.*^[126]^ 2016 Wingelhofer *et al.*^[127]^ 2018
Wnt/β-catenin	CBP/β-catenin CBP/β-catenin β-catenin/TCF β-catenin CK1α	Small molecule inhibitor Small molecule inhibitor Small molecule inhibitor Small molecule inhibitor Agonist	PRI-724 CWP232228 CWP232291 BC2059 Pyrvinium	Jiang *et al.*^[128]^ 2018 Benoit *et al.*^[129]^ 2017 Kim *et al.*^[101]^ 2011 Fiskus *et al.*^[130]^ 2015 Fong *et al.*^[131]^ 2015
Notch	Notch2 Notch1 Notch1/2 γ-secretase	Agonist Agonist Agonist GSI	Dll4-Fc NMHC AZA BMS-906024	Lobry *et al.*^[118]^ 2013 Ye *et al.*^[132]^ 2016 Dongdong *et al.*^[133]^ 2019 Arenas *et al.*^[134]^ 2018; Grieselhuber *et al.*^[119] ^2013
Hedgehog	Smoothened Smoothened Smoothened GLI1/2	Small molecule inhibitor Small molecule inhibitor Small molecule inhibitor Small molecule inhibitor	PF-913 Sonidegib Saridegib GANT-61	Fukushima *et al.*^[135]^ 2016 Li *et al.*^[136]^ 2016 Lim *et al.*^[137]^ 2015 Long *et al.*^[138]^ 2016
NF-κB	IKK NF-κB NF-κB	Small molecule inhibitor GSL NSAID	BMS-345541 Micheliolide CMT	Reikvam^[95]^ 2020 Ji *et al.*^[98]^ 2016 Strair *et al.*^[139]^ 2008
HDAC	HDAC HDAC HDAC HDAC	Benzamide-type inhibitor Pan inhibitor Pan inhibitor Benzamide-type inhibitor	Entinostat Panobinostat Pracinostat Chidamide	Zhou *et al.*^[140]^ 2013 Fiskus *et al.*^[130]^ 2015 Novotny-Diermayr *et al.*^[141]^ 2012 Li *et al.*^[142]^ 2015
*Other*				
ALDH	ALDH2 ALDH1/3	Non-specific inhibitor Competitive inhibitor	Disulfiram DIMATE	Yang *et al.*^[143]^ 2020 Venton *et al.*^[144]^ 2016
CXCR4	CXCR4 CXCR4 CXCR4 CXCR4	Small molecule inhibitor Small molecule inhibitor Small molecule inhibitor Monoclonal antibody	Plerixafor BL-8040 AMD3465 BMS-936564	Tavor *et al.*^[145]^ 2008 Abraham *et al.*^[146]^ 2017 Zeng *et al.*^[147] ^2009 Kuhne *et al.*^[148] ^2013

dODN: Decoy oligodeoxynucleotide; CBP: CREB-binding protein; Sam68: SRC-associated in mitosis 68; CK1α: casein kinase 1α; Dll4-Fc: delta-like 4 extracellular domain fused to the IgG-Fc-fragment; NMHC: *N*-methylhemeanthidine chloride; AZA: azelaic acid; GSI: gamma-secretase inhibitor; GLI: glioma-associated oncogene homolog; IKK: IκB kinase; GSL: guaianolide sesquiterpene lactone; CMT: choline magnesium trisalicylate; NSAID: non-steroidal anti-inflammatory drug; DIMATE: dimethyl ampal thiolester; HDAC: histone deacetylase; ALDH: aldehyde dehydrogenase.

## LEUKEMIC STEM CELLS IN ACUTE LYMPHOID LEUKEMIA

Although AML is proven to be maintained by a rare population of LSCs, for ALL it is not clear if it is organized similarly. Several studies support a similar hierarchical organization in ALL as AML, while other studies provide evidence that contradicts this.

### Leukemic stem cells in B-ALL

Two early studies from Cox *et al.*^[149,150]^ evaluated the long-term proliferation of childhood B-ALL cells *in vitro* and *in vivo*. The expression of several surface markers, such as CD34, CD38, CD19, CD133, and CD10, on the B-ALL cells were investigated on their potential to initiate B-ALL. Only a minority of B-ALL cells, those with a primitive CD34+/CD10-/CD19−/CD38- phenotype, were capable of engrafting B-ALL in NOD/SCID mice. Since CD19 is known to be a B-lymphocyte antigen and CD10 a marker of lymphocytic differentiation, this result suggests that B-ALL arises, as in AML, from a primitive immature cell instead of a committed B cell^[34,149]^. A few years later, this group performed a follow-up study on the expression of the primitive cell antigen CD133. They found that only cells within the CD133+/CD19- and CD133+/CD38- phenotypes were capable of initiating B-ALL in children, which supported their previous findings^[150,151]^.

Besides studies indicating that only cells with a primitive phenotype are able to engraft B-ALL in NOD/SCID mice, several other studies found contradictory results showing that exclusively the more mature CD19+ B-ALL cells were capable of engrafting^[152,153]^. In this latter study, both the CD34+/CD38-/CD19+ and the CD34+/CD38+/CD19+ B-ALL cell populations had the capacity to engraft B-ALL. Furthermore, in high-risk childhood ALL patients, including patients with *MLL* gene rearrangement, blasts cells were within three different maturation stages (CD34+CD19-, CD34+CD19+ and CD34-CD19+), which all had the capacity to re-establish and reconstitute the original leukemia phenotype in NOD/SCID mice^[154]^. That cells within different maturation stages have the capacity to engraft B-ALL and contain stem cell activity was confirmed by showing that both CD19+/CD20- and CD19+/CD20+ cells are capable of B-ALL engraftment. However, a study specifically looking at *MLL-AF4+* infant B-ALL showed that exclusively the more mature CD34+CD19+ and CD34-CD19+ B-ALL cell populations could engraft^[155]^. Similar results were seen in standard-risk patients, such as patients with a *TEL/AML1* fusion gene, in whom engraftment of B-ALL was restricted to cells containing the CD19+ phenotypes^[154]^. These controversial findings highlight that the LSCs in B-ALL are heterogeneous and indicate that several cytogenetic aberrations are involved in driving LSCs.

Since in AML the enrichment of LSCs is well established and the CD34+CD38- fraction is suggested to contain the most important LSCs^[17]^, the surface markers CD34 and CD38 have also often been used to enrich for LSCs in B-ALL^[156]^. However, there is a highly dynamic expression of CD34 and CD38 on leukemia-initiating cells in B-ALL^[157]^, which could be an explanation for the controversial results found in the above-mentioned studies and also suggests a non-hierarchical organization of B-ALL.

Taken together, accumulating evidence shows that there are several different ALL LSCs with a variety of immunophenotypes, making it impossible to isolate these cells based on their surface markers. Moreover, this indicates that there is not just a rare subset of B-ALL cells with an enhanced leukemogenic potential, and that the stochastic model, rather than the hierarchical LSC model observed in AML, applies for B-ALL^[33,158]^.

### Leukemic stem cells in T-ALL

The identification of LSCs in human T-ALL is, as in B-ALL, a major challenge. Cox *et al.*^[159]^ tried to identify LSCs in pediatric T-ALL patients by performing* in vitro* suspension culture assays and* in vivo* NOD/SCID mice model experiments. T-ALL cells with the expression of CD34, in combination with CD4 and CD7, were investigated for their potential to be LSCs. CD4 is a co-receptor of the T cell receptor and CD7 is a marker of early T cell differentiation^[160,161]^. Exclusively cells within the rare CD34+/CD4- and CD34+/CD7- subfractions were capable of T-ALL engraftment in mice, suggesting that pediatric T-ALL arises from cells with a primitive immunophenotype, and that T-ALL is similarly organized as in AML^[159]^. Contradicting results were found in a study that investigated cortical/mature T-ALL patient samples. In these T-ALL patients, the LSC activity was limited to cells within the CD34+/CD7+ subpopulation both *in vitro* and *in vivo*, while the primitive CD34+/CD7- cells only grew out into normal HSCs^[161]^.

The previous two studies suggest that LSC activity in T-ALL is limited to the CD34+ phenotype. However, it was revealed that the CD34+ fraction in some T-ALL samples also contained LSC activity, while in other samples the LSC activity was in the CD34- population^[162]^. This indicates that, as seen in B-ALL, CD34 is not a universal marker to identify LSCs in all adult T-ALL patients. Interestingly, Chiu *et al.*^[162]^ found that a CD7+/CD1a- T-ALL cell subset was enriched for LSC activity, suggesting that adult T-ALL arises from immature thymocytes and is organized as a hierarchical CSC model. Moreover, these CD7+/CD1a- cells were shown to be resistant to glucocorticoids such as dexamethasone and prednisone^[162]^. These drugs are commonly used for the treatment of ALL, and particularly in T-ALL, resistance to glucocorticoids is the most important driver of treatment failure^[163]^. Together, these results indicate that the CD7+/CD1a- T-ALL cell fraction is functionally different from the bulk of the T-ALL, and that it might be important to eliminate these cells to overcome treatment failure.

Besides studies investigating possible surface markers in T-ALL, there are also studies focusing on signaling pathways that could play a key role in T-ALL relapse. Since the majority of patients have T-ALL with oncogenic *Notch1* mutations, a recent study explored the significance of interleukin-7 receptor (IL-7R) signaling, a transcriptional target of Notch1, in LSC potential in T-ALL cell lines, human pediatric samples, and one adult T-ALL sample^[164]^. Expression of functional IL-7R is crucial for the emergence of Notch1-induced T-ALL, and IL-7R was demonstrated to be a biomarker for LSCs in T-ALL. Besides human T-ALL, IL-7R is also essential in B-ALL cells containing LSC activity and promoting B-ALL progression, suggesting that targeting IL-7R could prevent relapse in both pediatric T-ALL and B-ALL patients^[164]^.

Despite a less well-characterized ALL organization compared to AML, there are several clinical trials that focus on targeting populations of cells that have a high initiating potential in ALL such as CD34+CD38+CD19+ cells^[153]^. Besides these ALL trials, numerous clinical trials targeting AML LSCs are currently active.

## CLINICAL TRIALS TARGETING LSCs

Many different surface markers, transcription factors, and other factors have been described to be differentially expressed between HSCs and LSCs. These are all promising targets in the treatment of acute leukemia, and therefore currently, several clinical trials are investigating the effect of drugs targeting them. The clinical trials in Supplementary Table 1 are examples of recent clinical trials investigating the effects of these anti-LSC compounds, either as a monotherapy or in combination with other therapies.

### Summary and important remarks of recent clinical trials targeting LSC-specific markers

Supplementary Table 1 shows many clinical trials focusing on the markers mentioned in the previous sections. Most of these trials, investigating novel therapeutic compounds targeting AML LSCs, are phase I or II trials; however, some compounds are already in phase III: CD123/CLL-1 CAR-T cells, Hh pathway inhibitor PF-913, and HDAC inhibitors pracinostat and panobinostat. Besides the majority of these clinical trials focusing on LSCs in AML, a few have investigated compounds targeting cells capable of initiating B-ALL or T-ALL: anti-CD25 ADC ADCT-301, bispecific anti-CD19/CD3 BiTE blinatumomab, anti-CD19 CAR-T cells, anti-CD7 CAR-T cells, JAK1/2 inhibitor ruxolitinib, Notch inhibitor BMS-906024, and CXCR4 antagonists. The use of CAR-T cells is an often-used strategy to target surface markers in ALL as well as AML. Interestingly, the use of CAR-T cells is one of the few therapeutic strategies in clinical trials with patients under the age of 18. Clinical trials using other compounds or targeting LSC-related pathways are exclusively performed in adults.

Although some clinical trials are investigating the safety and efficacy of novel therapeutics as a monotherapy, most of the compounds are tested in combination with other drugs, such as chemotherapy and hypomethylating agents. A few clinical trials have been terminated [Supplementary Table 1], either due to a lack of efficacy or slow enrollment, but not because toxicities were seen. Despite this, many recent clinical trials are still recruiting and some clinical trials targeting LSC-related surface markers or pathways have even been completed with published results.

### Published results from recent clinical trials targeting LSC-specific markers

Results from two recent clinical trials involving the targeting of AML LSC surface markers have been published. The clinical trial evaluating the safety and efficacy of anti-CD33 (ADC SGN-CD33A) in newly diagnosed AML patients (NCT02326584) demonstrated that ADC SGN-CD33A is safe both as a monotherapy and in combination with standard high-dose cytarabine (HiDAC) therapy. As a single agent, it resulted in on-target myelosuppression with mild non-hematologic side effects when administered after chemotherapy and/or after allogeneic stem cell transplantation^[165]^. Besides CD33, the surface marker CD47 has been tested as an LSC target. Relapsed/refractory AML patients treated with the anti-CD47 monoclonal antibody Hu5F9-G4 (NCT02678338) showed decreased hemoglobin levels in all patients and difficulties with blood compatibility testing^[166]^. This could be explained by the fact that CD47 is also expressed on red blood cells, suggesting that HU5F0-G4 is capable of clearing these red blood cells. This therapeutic can only be used safely by carefully monitoring patients receiving Hu5F9-G4.

**Figure 2:**
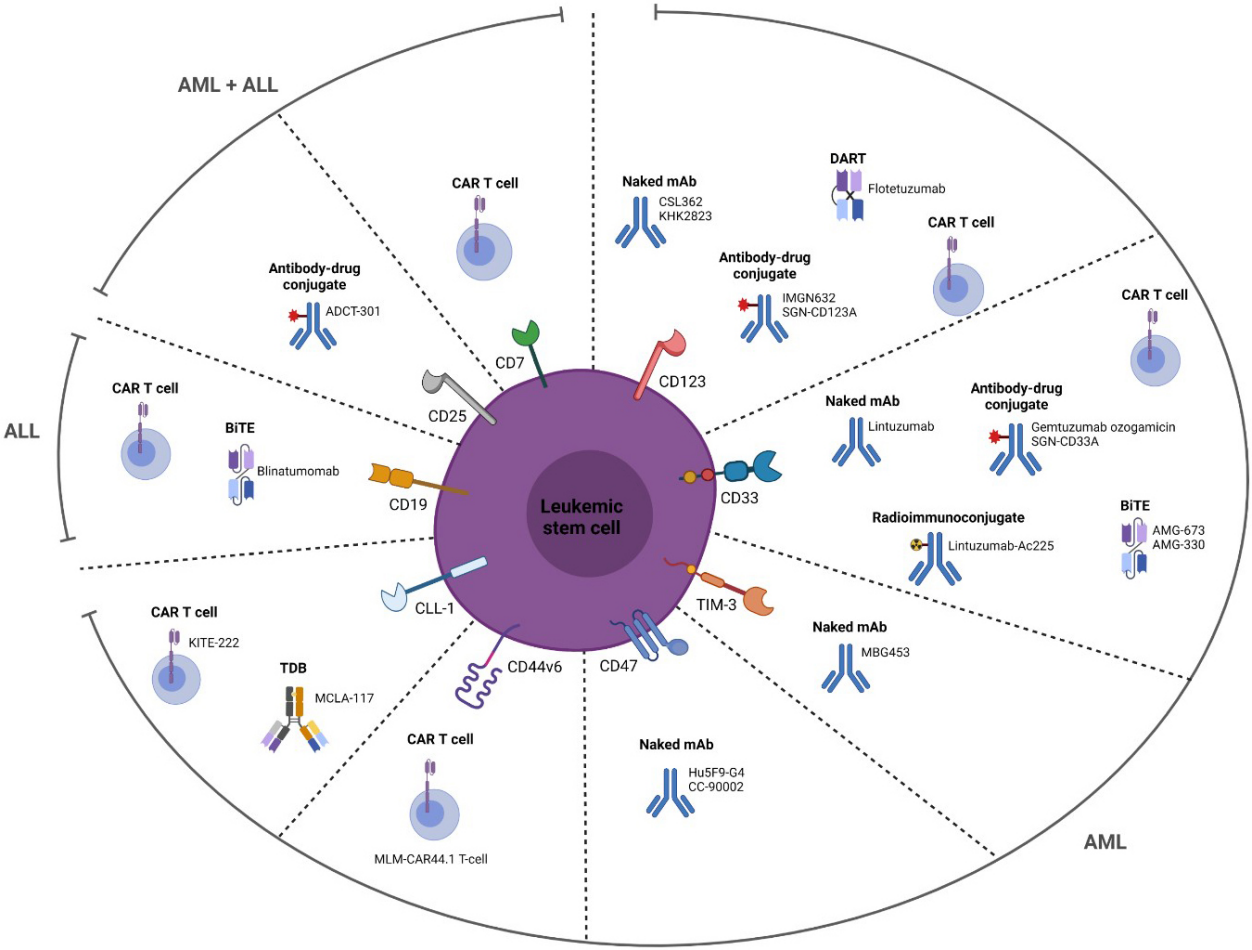
LSC-specific surface markers and the different types of drug agents targeting these markers. All depicted surface markers except CD19 are differentially expressed on AML LSCs. CD19 is a marker specific for B-ALL blasts. In addition, CD7 and CD25 have also been used as targets for ALL treatment. The depicted agents have recently been used in clinical trials. Created with BioRender.com. LSC: Leukemic stem cell; AML: acute myeloid leukemia; CAR: chimeric antigen receptor; TDB: T cell-dependent bispecific; DART: dual-affinity re-targeting antibody.

To investigate the effect of targeting CD19+ B-ALL blasts, blinatumomab, a bispecific anti-CD19/CD3 BiTE, has been used in multiple clinical trials. Results from a phase III clinical trial (NCT02013167) have shown that, compared to chemotherapy, blinatumomab results in improved minimal residual disease remission and longer overall survival in adult B-ALL patients^[167,168]^, suggestive for eradicating B-ALL LSCs. In addition, a recent study (NCT02143414) showed benefits for older patients with newly diagnosed Ph chromosome-negative B-ALL, including patients with poor-risk cytogenetics^[169]^. Besides antibody therapy, CAR-T therapy targeting CD19 has been shown to be effective in B-ALL^[170,171]^, although it is not specifically shown to be particularly directed against LSCs. An overview of all recent therapeutic strategies targeting LSC-specific surface markers in both AML and ALL is shown in Figure 2.

In addition to clinical trials investigating drugs against the LSC surface markers, results from several clinical trials targeting LSC-related signaling pathways [Figure 1] have been published. First, two JAK inhibitors, ruxolitinib and pacritinib, showed promising results. Patients with relapsed/refractory AML treated with ruxolitinib (NCT00674479) revealed that this JAK inhibitor has limited toxicities and modest anti-leukemic activity^[172]^. Combining the JAK inhibitor pacritinib with chemotherapy in AML patients with *FLT3* mutations (NCT02323607) gave similar results as ruxolitinib, since it was well tolerated and showed anti-leukemic activity^[173,174]^. Second, Hh pathway inhibitors, such as PF-913 and sonidegib targeting Smoothened, have been investigated. In a phase II study (NCT01546038), the combination of PF-913 and low-dose cytarabine improved the overall survival of AML patients when compared to low-dose cytarabine alone. This improved outcome was predominantly seen in patients with secondary AML^[175]^. Notably, sonidegib combined with the hypomethylating agent azacitidine (NCT02129101) did not show such an improvement, compared to single azacitidine treatment, in patients with previously treated or advanced myeloid malignancies. However, in the relapsed/refractory AML patient population, the combination of sonidegib with azacitidine showed an increase in OS rates and an absence of progression^[176]^. Finally, the Wnt/β-catenin pathway inhibitor CWP232291 has been investigated as a single agent in relapsed/refractory AML patients (NCT01398462). This inhibitor was well tolerated but only minimally effective^[173]^. Despite this disappointing result, CWP232291 has great potential in the elimination of LSCs when used in combination with other drugs such as chemotherapy, since this Wnt/β-catenin pathway inhibitor may be very efficient in killing leukemia cells with self-renewal potential such as LSCs.

## DISCUSSION

The substantial number of recent studies investigating an LSC population in AML has led to the general acceptance of AML being hierarchically organized. The rare population of LSCs at the apex of this hierarchy plays a key role in the relapse development of the disease due to their drug-resistant mechanisms and self-renewal capacity^[17]^. Several common surface markers have been identified that enable both the specific eradication and isolation for further analysis of this LSC population [Table 1]. In addition, different pathways regulating LSCs “stemness” are often dysregulated, making them highly promising targets for the elimination of these cells [Table 2]. The focus of current clinical trials is on novel therapies targeting LSC specific markers by using immunotherapy-based drugs such as CAR-T cells directed to LSC specific surface markers or small-molecule inhibitors/agonists directed to “stemness” pathways [Supplementary Table 1]. However, the multiple resistance mechanisms contained by LSCs are a major challenge in achieving an effective eradication of these cells. Therefore, many clinical trials in AML patients test the effectiveness of targeting LSCs using several therapies, both as a monotherapy and in combination with other drugs.

Accumulating evidence shows that the interaction between BM niche and LSCs plays a role in the survival and acquired drug resistance of AML LSCs^[177]^. For instance, inhibiting the CXCR4/SDF1 interaction involved in HSC/LSC homing releases LSCs from their chemoprotective niche, making them more sensitive to chemotherapy^[30]^. This indicates that, besides specifically targeting LSCs, targeting the pathways involved in the BM/LSC interaction could also be a useful strategy to increase LSC eradication. However, this strategy has to be executed carefully, since releasing leukemic cells from the BM into the peripheral circulation could possibly increase the risk of these blasts infiltrating other organs. Future studies on AML should therefore focus on gaining better insight into the factors involved in LSC homing and the effectiveness and safety of targeting these factors.

Compared to AML, research on the LSC population in ALL has been less successful with highly contradicting results. When comparing AML with T-ALL, pathophysiological similarities are seen within both diseases^[162,178]^. However, it is still not fully understood if T-ALL follows the same stem cell model as AML. The majority of B-ALL cells containing different immunophenotypes have the capacity to initiate ALL, indicating that B-ALL lacks a clear hierarchy as in AML^[158]^. Hence, the term LSC as used in AML might not be correct to define the cells being able to cause relapse in ALL. For ALL, the term leukemia-initiating cells (LICs) is more preferred. Due to the dynamic phenotypes of LICs, their prospective purification has not been possible up to now^[33]^. More research is needed to investigate ALL LIC plasticity and discriminating factors that make one population of cells more likely to result in relapse than the other. However, since this has shown to be highly challenging, the focus on interfering with the leukemic cells and BM interaction could be a more promising approach to eliminate ALL LICs in the near future. For all these novel LSC-targeted agents, it is difficult to assess whether specifically the LSC are really eliminated. Monitoring the LSC using flow cytometry during and after therapy might be an option^[179,180]^ but is not standardly investigated yet.

Despite many different surfaces and other markers being promising targets in eradicating LSCs, there are some important considerations that need to be taken into account before applying anti-LSC therapeutics. First, it is extremely important that healthy cells, especially HSCs, are not targeted by the treatment. Since some surface markers and intracellular pathways involved in “stemness” are expressed in LSCs and HSCs, there is a concern that targeting LSCs can harm healthy stem cell populations. This might cause severe and dangerous side effects, so more research is needed to evaluate how many healthy cells are affected in patients treated with these therapies. Secondly, a major challenge is the heterogeneity of acute leukemia. For instance, in AML, heterogeneous phenotypes of LSCs have been identified between patients^[181]^. In addition, it is possible that there are multiple populations of LSCs with different phenotypes present within one patient. Targeting only one marker may therefore not be effective, and more research focusing on simultaneously or subsequently targeting multiple markers is needed.

## DECLARATIONS

### Availability of data and materials

This review article was based on previously published research and reviews, which were collected by using a search of the literature, and they are cited in the review.

### Copyright

© The Author(s) 2022.
