# Supplementary material for: Characteristics of leukemic stem cells in acute leukemia and potential targeted therapies for their specific eradication
Source: Cancer Drug Resist. 2022 May 5;5(2):344–67. doi: 10.20517/cdr.2021.140 (PMC9255252; doi:10.20517/cdr.2021.140)
Supplement: Supplementary file 1 [file cdr-5-2-344-SupplementaryMaterials.pdf]

Supplementary Table 1. Examples of clinical trials targeting leukemia initiating cells in AML and ALL (currently ongoing or completed/terminated between 2013 and 2021)

Clinical trials	Disease type	Target group	Intervention	Phase	Status
LSC specific surface markers					
NCT02992860	AML and MDS	R/R AML and MDS (age ≥ 18 years)	Anti-CD123 monoclonal antibody (CSL362)	II	Terminated*
NCT02181699	AML and MDS	R/R AML and MDS or previously untreated AML (age ≥ 18 years)	Anti-CD123 monoclonal antibody (KHK2823)	I	Terminated**
NCT02848248	AML	R/R AML (age 18-74 years)	Anti-CD123 ADC (SGN-CD123A)	I	Terminated***
NCT04086264	AML	CD123+ AML (age ≥ 18 years)	Anti-CD123 ADC (IMGN632) monotherapy or combined with venetoclax and/or azacitidine	I/II	Recruiting
NCT04158739	AML	R/R AML (age ≤ 20 years)	Cytarabine combined with CD3/CD123 DART (Flotetuzumab)	I	Recruiting
NCT03066648	AML and MDS	R/R or newly diagnosed AML and high risk MDS (age ≥ 18 years)	Anti-TIM-3 monoclonal antibody (MBG453) monotherapy or combined with decitabine and/or anti-PD-1 antibody (PDR001)	I	Recruiting
NCT04219163	AML	R/R AML (age ≤ 74 years)	CLL-1.CAR-T cells	I	Recruiting
NCT03795779	AML	High risk R/R AML (all ages)	CLL-1-CD33 cCAR-T cells	I	Recruiting

NCT03631576	AML	R/R AML (age ≤ 70 years)	CD123/CLL-1 CAR-T cells	II/III	Recruiting
NCT04010877	AML	R/R AML (age 6 months to 75 years)	CLL-1, CD33 and/or CD123 CAR-T cells	I/II	Recruiting
NCT03878927	AML	Non-intensive treated AML (age ≥ 55 years)	Cytarabine: daunorubicin (CPX-351) + anti-CD33 ADC (gemtuzumab ozogamicin)	I	Recruiting
NCT02326584	AML	Newly diagnosed AML (age ≥ 18 years)	Anti-CD33 ADC (SGN-CD33A) monotherapy or combined with standard-of-care	I	Completed
NCT03441048	AML	R/R AML (age ≥ 18 years)	CLAG-M chemotherapy combined with anti-CD33 radioimmunoconjugate (Lintuzumab-Ac225)	I	Recruiting
NCT03224819	AML	R/R AML (age ≥ 18 years)	CD3/CD33 BiTE (AMG-673)	I	Active, not recruiting
NCT03038230	AML	R/R AML (age ≥ 18 years) or newly diagnosed untreated high-risk elderly	CD3/CLL-1 bispecific antibody (MCLA-117)	I	Unknown
NCT04789408	AML	R/R AML (age ≥ 18 years)	Cyclophosphamide and fludarabine + CLL-1 CAR-T cells (KITE-222)	I	Not yet recruiting
NCT02588092	AML and ALL	R/R CD25+ AML or ALL (age ≥ 18 years)	Anti-CD25 ADC (ADCT-301)	I	Terminated****

NCT04097301	AML and MM	AML or MM expressing CD44v6 (age 1–75 years)	CD44v6 CAR-T cells (MLM-CAR44.1 T-cells)	I/II	Recruiting
NCT02678338	AML and MDS	R/R AML or high risk MDS (age ≥ 18 years)	Anti-CD47 monoclonal antibody (Hu5F9-G4)	I	Completed
NCT02641002	AML and MDS	R/R AML or MDS (age ≥ 18 years)	Anti-CD47 monoclonal antibody (CC-90002)	I	Terminated*****
NCT02879695	B-ALL and MPAL	Poor risk R/R CD19+ precursor B-ALL (age ≥ 16 years)	PD-1 inhibitor (nivolumab) and/or CTLA-4 inhibitor (ipilimumab) + bispecific anti-CD19/CD3 BiTE (blinatumomab)	I	Recruiting
NCT04524455	B-ALL	R/R precursor B-ALL (age ≥ 18 years)	Anti-PD-1 monoclonal antibody (AMG404) + bispecific anti-CD19/CD3 BiTE (blinatumomab)	I	Recruiting
NCT04778579	ALL	R/R CD19+ ALL (age 18-70 years)	Anti-CD19 CAR-T cells (ARI-0001)	II	Not yet recruiting
NCT03144583	ALL, CLL, lymphoma	R/R CD19+ malignancies (age 2–80 years)	Anti-CD19 CAR-T cells (ARI-0001	I	Active, not recruiting
NCT02435849	B-ALL	R/R CD19+ B-ALL (age ≤ 25 years)	Anti-CD19 CAR-T cells (CTL019)	II	Active, not recruiting
NCT04033302	AML, T-ALL, T-LBL and NKL	CD7+ AML, T-ALL, T-LBL or NKL (age 6 months to 75 years)	Anti-CD7 CAR-T cells	I/II	Recruiting

NCT04762485	AML, T-ALL and T-LBL	R/R CD7+ AML, T-ALL or T-LBL (age 12-65 years)	Anti-CD7 CAR-T cells	I/II	Not yet recruiting
NCT04620655	T-ALL and T-LBL	R/R CD7+ T-ALL or T-LBL (age 3-70 years)	Anti-CD7 CAR-T cells (RD13-01)	Not applicable	Recruiting
LSC-related signaling pathways					
NCT00674479	AML, MDS, ALL and CML	R/R advanced hematological malignancies (age ≥ 18 years)	JAK1/2 inhibitor ruxolitinib (INCB018424)	II	Completed
NCT02257138	AML and MDS/MPN	R/R or post myeloproliferative AML (age ≥ 18 years)	Decitabine + JAK1/2 inhibitor (ruxolitinib phosphate)	I/II	Active, not recruiting
NCT02323607	AML	AML with *FLT3* mutation (age ≥ 18 years)	Cytarabine and daunorubicin hydrochloride or decitabine + JAK2 inhibitor (pacritinib)	I	Completed
NCT03063944	AML	Refractory AML or AML ineligible for intensive chemotherapy (age ≥ 18 years)	Decitabine + venetoclax + small molecule STAT3 inhibitor (OPB-111077)	I	Recruiting
NCT01363817	T-ALL and T-LBL	R/R T-ALL or T-LBL (age ≥ 18 years)	Dexamethasone + small molecule Notch inhibitor (BMS-906024)	I	Completed
NCT01546038	AML and MDS	AML or high-risk myelodysplastic syndrome	Intensive chemotherapy, low dose ARA-C or decitabine + small molecule Hh	II	Completed

		(age ≥ 18 years)	pathway inhibitor (PF-913)		
NCT03416179	AML	Untreated or secondary AML (age ≥ 18 years)	Azacitidine or cytarabine and daunorubicin + small molecule Hh pathway inhibitor (PF-913)	III	Active, not recruiting
NCT02129101	AML CML, MDS, CMML and MPN	Myeloid malignancies including R/R or untreated AML (age ≥ 18 years)	Azacitidine + decitabine or small molecule Hh pathway inhibitor (sonidegib)	I	Completed
NCT01606579	AML, MDS and CML	Advanced myeloid malignancies including R/R AML (age ≥ 18 years)	Low dose ARA-C or dasatinib + small molecule Wnt/β-catenin pathway inhibitor (PRI-724)	I/II	Completed
NCT01398462	AML, CMML-2 and MDS	Myeloid malignancies including R/R AML (age ≥ 18 years)	Small molecule Wnt/β-catenin pathway inhibitor (CWP232291)	I	Completed
NCT02144675	AML	AML (age ≥ 18 years)	Idarubicin and cytarabine + NF-κB inhibitor (CMT)	II	Completed
NCT03151408	AML	Newly diagnosed AML (age ≥ 18 years)	Azacitidine + small molecule HDAC inhibitor (pracinostat)	III	Terminated******
NCT00313586	MDS, CMML and AML	De novo MDS, dysplastic CMML or trilineage dysplasia AML (age ≥ 18	Azacitidine + small molecule HDAC inhibitor (entinostat)	II	Completed

		years)			
NCT04326764	AML and MDS	High risk AML or MDS having received allogeneic HSCT (age 18-70 years)	Standard of care or HDAC inhibitor (panobinostat) maintenance therapy	III	Recruiting
Other LSC-related markers					
NCT01319864	AML, ALL, MDS	R/R AML/ALL or secondary AML/MDS (age 3 to 29 years)	Cytarabine and etoposide + CXCR4 antagonist (plerixafor)	I	Completed
NCT02763384	T-ALL	R/R T-ALL (age ≥ 18 years)	Nelarabine + CXCR4 antagonist (BL-8040)	II	Recruiting

*Terminated due to a parallel clinical trial with TALA that showed no efficacy. **Terminated due to failed treatment response. ***Reason of termination unknown. ****Terminated due to slow enrollment. *****Terminated due to insufficient encouraging profile in preliminary monotherapy data. ******Terminated due to lack of efficacy. R/R: Refractory/relapsed; AML: acute myeloid leukemia; MDS: myelodysplastic syndrome; ALL: acute lymphoid leukemia; CLL: chronic lymphoid leukemia; CML: chronic myeloid leukemia; CMML: chronic myelomonocytic leukemia; MPAL: mixed phenotype acute leukemia; ADC: antibody-drug conjugate; CAR: chimeric antigen receptor; cCAR: compound chimeric antigen receptor; BiTE: bispecific T-cell engager; NKL: natural killer cell lymphoma; T-LBL: T-cell lymphoblastic lymphoma; Hh: Hedgehog; CMT: choline magnesium trisalicylate; HSCT: hematopoietic stem cell transplantation.
